# Impact of sublethal exposure to a pyrethroid-neonicotinoid insecticide on mating, fecundity and development in the bed bug *Cimex lectularius* L. (Hemiptera: Cimicidae)

**DOI:** 10.1371/journal.pone.0177410

**Published:** 2017-05-10

**Authors:** Sydney E. Crawley, Jennifer R. Gordon, Katelyn A. Kowles, Michael F. Potter, Kenneth F. Haynes

**Affiliations:** Department of Entomology, University of Kentucky, Lexington, KY, United States of America; Institut Sophia Agrobiotech, FRANCE

## Abstract

Sublethal exposure to an insecticide may alter insect feeding, mating, oviposition, fecundity, development, and many other life history parameters. Such effects may have population-level consequences that are not apparent in traditional dose-mortality evaluations. Earlier, we found that a routinely used combination insecticide that includes a pyrethroid and a neonicotinoid (Temprid^®^ SC) had deleterious effects on multiple bed bug (*Cimex lectularius*, L.) behaviors. Here, we demonstrate that sublethal exposure impacts physiology and reproduction as well. We report that sublethal exposure to Temprid SC has variable aberrant effects on bed bugs depending on the strain, including: a reduction in male mating success and delayed oviposition by females. However, after sublethal exposure, egg hatch rate consistently declined in every strain tested, anywhere from 34%-73%. Conversely, impact on fifth instar eclosion time was not significant. While the strains that we tested varied in their respective magnitude of sublethal effects, taken together, these effects could reduce bed bug population growth. These changes in bed bug behavior and fecundity could lead to improved efficacy of Temprid SC in the field, but recovery of impacted bugs must be considered in future studies. Sublethal effects should not be overlooked when evaluating insecticide efficacy, as it is likely that other products may also have indirect effects on population dynamics that could either aid or inhibit successful management of pest populations.

## Introduction

Sublethal exposure to an insecticide may affect many life history parameters for insects, including: reductions in feeding or searching time, diminished life span, alterations in development time, and diminished mating and/or fecundity [1–6]. Declines in fecundity may occur through a variety of mechanisms, including alterations of spermatogenesis, sperm motility, oogenesis, ovulation, or egg fertilization after exposure [7]. Changes in life span, development, mating, fecundity, and feeding after sublethal exposure may have important population-level consequences [8]. The evaluation of insecticide efficacy based solely on acute toxicity, which is a standard practice, might overlook population level consequences caused by sublethal effects. Applying the information gleaned from standardized acute toxicity laboratory assays at the population-level can be problematic if there are large differences in the insecticide concentrations or exposure times leading to lethal versus sublethal effects [5]. Thus, there is the potential for a substantial gap in our knowledge of the sublethal effects of insecticide exposure for many arthropod pest populations. Agricultural systems are an exception, where there has been interest in the sublethal effects of insecticides on beneficial species such as the honey bee, *Apis mellifera*, non-target arthropods, and the major agricultural pests causing economic loss [1,9,10–12]. However, there are far fewer studies on sublethal effects for urban and household pests. Bed bugs (*Cimex lectularius*, L.) are an urban pest that is becoming increasingly difficult to manage due to widespread insecticide resistance [13–16]. Since insecticides remain the most common tool for treating infestations [17], understanding both lethal and sublethal effects of pesticides on populations is important.

Bed bugs (*C*. *lectularius*) are flightless, hematophagous insects with public health relevance since all life stages feed on human blood [18]. Their bites often cause itchy, painful wheals and occasionally more severe reactions [19, 20]. Additionally, infestations of bed bugs can have negative psychological outcomes from anxiety to depression, and even suicidal thoughts [21]. Because bed bugs are cryptic, and the majority of their activity occurs at night when the host is sleeping [22], they are hard to detect until an infestation is well established [23]. Missed bugs or survivors of insecticidal or non-chemical approaches could lead to further population growth and treatment failure. Thus, many pest management professionals prefer insecticides with residual activity to deter reinfestation.

Many recent studies have focused on insecticide resistance to pyrethroids and combination products [24,25]. However, very few studies have identified sublethal effects that could lead to various effects on bed bug populations. Two studies have explored the sublethal effects of ActiveGuard™ (permethrin-impregnated fabric) on bed bugs; exposure to the fabric alters behavior and negatively impacts their feeding and fecundity [26,27]. For instance, significantly fewer females laid eggs after sublethal exposure to ActiveGuard, and exposure times as short as one minute were enough to induce a decline in fecundity and feeding attempts [27]. These results indicate a potential for detrimental population-level consequences due to sublethal exposure. In a previous study, we demonstrated a negative impact of sublethal exposure to Temprid SC, the most widely used insecticide for bed bug management in the USA, on bed bug feeding and locomotion, but not on aggregation in daytime harborages [28]. Here we expand on those behavioral studies by addressing whether sublethal exposure to Temprid SC affects mating efficacy, as well as physiological parameters such as bed bug fecundity and development. Temprid SC contains two active ingredients, imidacloprid (a neonicotinoid), and β-cyfluthrin (a pyrethroid). We do not attempt to separate the individual impacts of these components; instead we examine the overall impact of this combination product as it is labeled for use. We hypothesized that at sublethal exposure times, treated insects would show decreased mating success, a reduction in fecundity, and prolonged development.

## Materials and methods

### Insects

Three strains of bed bugs (*Cimex lectularius*, L.) were used in each experiment. The progenitors of the CIN-1 colony were collected from Cincinnati, OH in 2005. The original colony was highly resistant to pyrethroids, but after generations in culture is now more susceptible [13]. NY-1 was collected from New York City, NY in 2007 and is moderately resistant to pyrethroids [13]. LEX-8 was collected from Lexington, KY in 2012, and is resistant to pyrethroid insecticides [16]. Pest management professionals collected insects from apartments during routine treatments. Permission was granted by landlords and tenants for collection. Bed bugs are not a protected or endangered species.

Bugs were reared in an incubator (Percival Scientific, Perry, IA) (27°C, 70% RH, 14:10 L:D) and weekly blood meals were administered with an artificial blood feeding system [29]. In this system, defibrinated rabbit blood (HemoStat, Dixon, CA and Quad Five, Rygate, MT) was delivered into glass mosquito feeders (Kimble Chase Custom Glass Shop, Vineland, NJ) and heated to 39° C with a circulating water bath. Parafilm lined the bottom of the mosquito feeder, and served as a barrier between bugs and the blood. Bed bugs contained in 59 mL plastic jars (Consolidated Plastics, Stow, OH) sealed with organza had to pierce the organza and the parafilm membrane to feed. Each adult was seven days past eclosion and had not yet taken a blood meal as adults when exposed to Temprid SC (unless stated otherwise).

### Residual deposit mortality bioassays

An LT_10_ (lethal time of exposure resulting in 10% mortality) was determined independently for each strain using a residual deposit bioassay [13,28]. Adult bed bugs were held individually in 24-well plates (Costar, Corning, NY) with wells measuring 1.6 cm in diameter. Each well was lined with Whatman^®^ #2 filter paper (Whatman, Maidstone, England) cut to a diameter of 1.7 cm. Each filter paper was saturated with 50 μL (0.075% a.i., with a 2:1 ratio of imidacloprid, and β- cyfluthrin) of the combination product diluted in water at the label rate, or water alone (as a control). Filter papers were given 24 hours to air dry prior to the mortality bioassay. At this time, a bed bug was confined to the treated surface in one well and scored for mortality at 5, 15, and 30 minutes; 1, 4, 12, and 24 hours; 3, 7 and 14 days (if necessary). Mortality was scored at each time point by gently turning bugs onto their dorsal side with soft forceps. If the insect could not recover by turning over to the ventral side, it was considered moribund. Three replicates with 10 bugs per replicate were conducted. These methods are identical to those previously described [28].

### Mating

We were interested in whether sublethal exposure impacts mating behavior in such a way that the fecundity of adults may be reduced. We performed two experiments to test the relative mating success of treated versus untreated bed bugs. Our first copulation choice test was conducted to determine whether females exposed to Temprid SC at the LT_10_ had an altered or reduced mating frequency compared to untreated females (when presented with an untreated male). The second, reciprocal choice test examined the same effect for treated versus untreated males (when presented with an untreated female). For this experiment, we defined successful copulation (or a copulatory event) as prolonged mounting of a female by the male, with curving of the male’s abdomen. Each experiment was replicated fifteen times. There were no instances in which copulation did not occur (i.e. no non-responders). The insects that copulated successfully were recorded.

### Fecundity with sublethal exposure before mating

This experiment was designed to examine fecundity of mixed mating pairs of bed bugs after sublethal exposure (LT10) to the label rate of Temprid SC, with exposure occurring prior to mating. Males and females were separated from one another shortly after adult eclosion. At seven days post-emergence they were exposed to either water (control) or the combination insecticide (treatment). Bed bugs from each strain were randomly assigned to one of four mating pairs: a control female with a control male, a control female with a treated male, a treated female with a control male, and a treated female with a treated male (complete factorial). Each pair was placed in a 59 mL plastic jar covered with organza, fed with rabbit blood once per week, and the numbers of viable offspring (hatched eggs) were counted after six weeks. This experiment was replicated eight times.

Because there was a short period when unfed teneral adult males and females were housed together before the treatment occurred, we repeated this experiment using insects that were never exposed to the opposite sex as adults to ensure that no mating events had occurred before pairing (although mating in unfed teneral adults are not likely) [30]. Only the control female/control male and treated female/treated male pairs were reevaluated using this approach. To obtain bed bugs with ensured virginity, we placed fifth instars that had recently fed into individual wells of a 96-well plate (Costar, Corning, NY) until eclosion. Seven days after these adults had emerged they were exposed to treatment or control condition for the LT_10_. At 24 hours after the initiation of exposure, healthy survivors were assigned to treatment. Each pair was placed in a 59 mL plastic jar covered with organza, fed with rabbit blood once per week, and the numbers of nymphs (and thus the number of eggs that hatched) were counted after six weeks. This experiment was replicated eight times.

### Fecundity with sublethal exposure after mating

Prior to the start of this experiment, two day old virgin adult bed bugs were permitted to take a blood meal. After feeding, engorged bugs were removed, and males and females were placed in 59 mL plastic jars covered with organza for three days to allow mating to occur. On day three, when they were five days post emergence, we exposed only adult females to their strain-specific sublethal dose of Temprid SC, or to water. After 24 hours, surviving female bed bugs were placed individually into wells of a 24-well plate (Costar, Corning, NY) lined with Whatman^®^ filter paper (Sigma-Aldrich, St. Louis, MO). Once per day (at the same time each day) for ten days, the numbers of eggs laid by each female were recorded. In addition, we recorded the number of eclosed first instars each day. Every 24 hours, the female was moved to an unoccupied well of a 24-well plate for accurate counts of egg germination time. Females were not fed again throughout the course of this experiment. Each female was followed until she stopped laying eggs, and until all eggs had sufficient time to hatch. Thus this assay gives information on the number of eggs laid, the time course of egg-laying, the timing of egg hatch and how these variables are affected by sublethal exposure to Temprid SC. Twelve replicates were performed for each strain.

### Development

This experiment was conducted to test whether sublethal exposure (LT10) to the label rate of Temprid SC affected development time from fifth instar to adult. Directly following exposure (at two days post molt), juvenile bed bugs were kept in an incubator for recovery (27°C, 70% RH, 14:10 L:D). One day after exposure, three day old control and treatment fifth instar bed bugs were permitted to take a blood meal from the artificial feeding system. Juveniles that took a blood meal were placed in individual wells of a 96-well plate (Costar, Corning, NY) and monitored until molting occurred. Because we only used juveniles that had taken a full blood meal, any impact of the insecticide on feeding was excluded. The day of molting was recorded. Fifteen replicates of this experiment were conducted per strain.

### Data analysis

Probit analysis was used for the calculation of strain-specific LT_10_ values [Minitab^®^ 15] [31]. Binomial tests were used to evaluate differences in mating success in the behavioral mating assay [Statistix 10.0] [32]. A one-way analysis of variance (ANOVA) was used to compare the number of hatched eggs from the fecundity assay where multiple mating pairs were tested [Statistix 10.0] [32]. All count data were square-root transformed prior to analysis because the original values were not normally distributed. Paired t-tests were used to analyze data from the fecundity assay when only two mating pairs were compared [Statistix 10.0.] [32]. A two sample t-test was used to look for differences in development time between insects exposed to the combination product versus those exposed to water [Statistix 10.0] [32]. Because strain-specific exposure times to insecticide were used, all analyses were conducted only within strain.

## Results and discussion

### Residual deposit mortality bioassays

As expected, the LT_10_ values for bed bugs exposed to Temprid SC at the label rate(0.075% a.i., with a 2:1 ratio of imidacloprid, and β- cyfluthrin) differed depending on the initial level of pyrethroid resistance for each strain. The LT_10_ values were 0.95 ± 0.65, 1.0 ± 0.48, and 5.0 ± 1.98 hours (mean ± s.e.m.) for CIN-1, NY-1, and LEX-8 adults, respectively. All subsequent experiments using adult bed bugs were performed using these LT_10_’s. These values have been previously reported [28]. Because strains may revert back to susceptibility as they are maintained in the laboratory, we also re-evaluated the LT_10_ values during the course of experiments. The LT_10_ values obtained from a secondary evaluation were 0.45 ± 0.14, 0.90 ± 0.33, and 4.35 ± 1.30 hours (mean ± s.e.m.) for CIN-1, NY-1, and LEX-8 adults, respectively. The very close association between the data in the two separate mortality bioassays supported our decision to use the first (and previously tested) set of exposure times in all current experiments.

LT_10_ values were determined independently for fifth instar bed bugs of each strain. The LT_10_ values were 0.98 ± 0.22, 0.97 ± 0.30, and 3.33 ± 1.02 hours (mean ± s.e.m.) for CIN-1, NY-1, and LEX-8, respectively. These data, for both adults and juveniles, are summarized in Table 1.

**Table 1:** Probit analysis of residual exposure of three populations of bed bugs to a combination product containing β-cyfluthrin and imidacloprid.

Strain^*a*^	Status^*b*^^,^^*c*^	LT_10_ (95% C.I.)	LT_50_ (95% C.I.)	Slope (± SEM)
**CIN-1**	**Adult 2014**^***d***^	0.95 (0.46–1.57)	8.43 (5.64–13.07)	0.59 ± 0.07
**CIN-1**	**Adult 2015**	0.45 (0.21–0.77)	5.37 (3.68–8.03)	0.52 ± 0.06
**CIN-1**	**Nymph 2015**	0.98 (0.58–1.44)	4.41 (3.29–5.88)	0.85 ± 0.09
**NY-1**	**Adult 2014**^***d***^	1.14 (0.43–2.11)	29.42 (16.48–70.91)	0.39 ± 0.06
**NY-1**	**Adult 2015**	0.90 (0.36–1.64)	19.13 (11.57–38.29)	0.42 ± 0.06
**NY-1**	**Nymph 2015**	0.97 (0.46–1.63)	10.10 (7.47–17.21)	0.53 ± 0.07
**LEX-8**	**Adult 2014**^***d***^	5.00 (1.90–8.93)	64.15 (34.26–200.29)	0.50 ± 0.10
**LEX-8**	**Adult 2015**	4.35 (1.95–7.09)	45.39 (27.31–106.79)	0.55 ± 0.10
**LEX-8**	**Nymph 2015**	3.33 (1.48–5.50)	37.52 (23.05–80.87)	0.53 ± 0.09

Each of the three bed bug strains were exposed to Temprid SC at the label rate (50 μL, 0.075% a.i., with a 2:1 ratio of imidacloprid, and β- cyfluthrin). Lethal times (h) and 95% fiducial confidence intervals and slopes are shown.

^*a*^ 30 bed bugs were tested per strain using previous methods [13].

^*b*^ Two assays were performed to ensure consistency in resistance status throughout time course of experiments.

^*c*^ 5^th^ instars represent ‘nymphs’ and were evaluated using the same methods as adult bed bugs.

^*d*^ Values and methods also listed in reference 28.

### Mating

**Fig 1:**
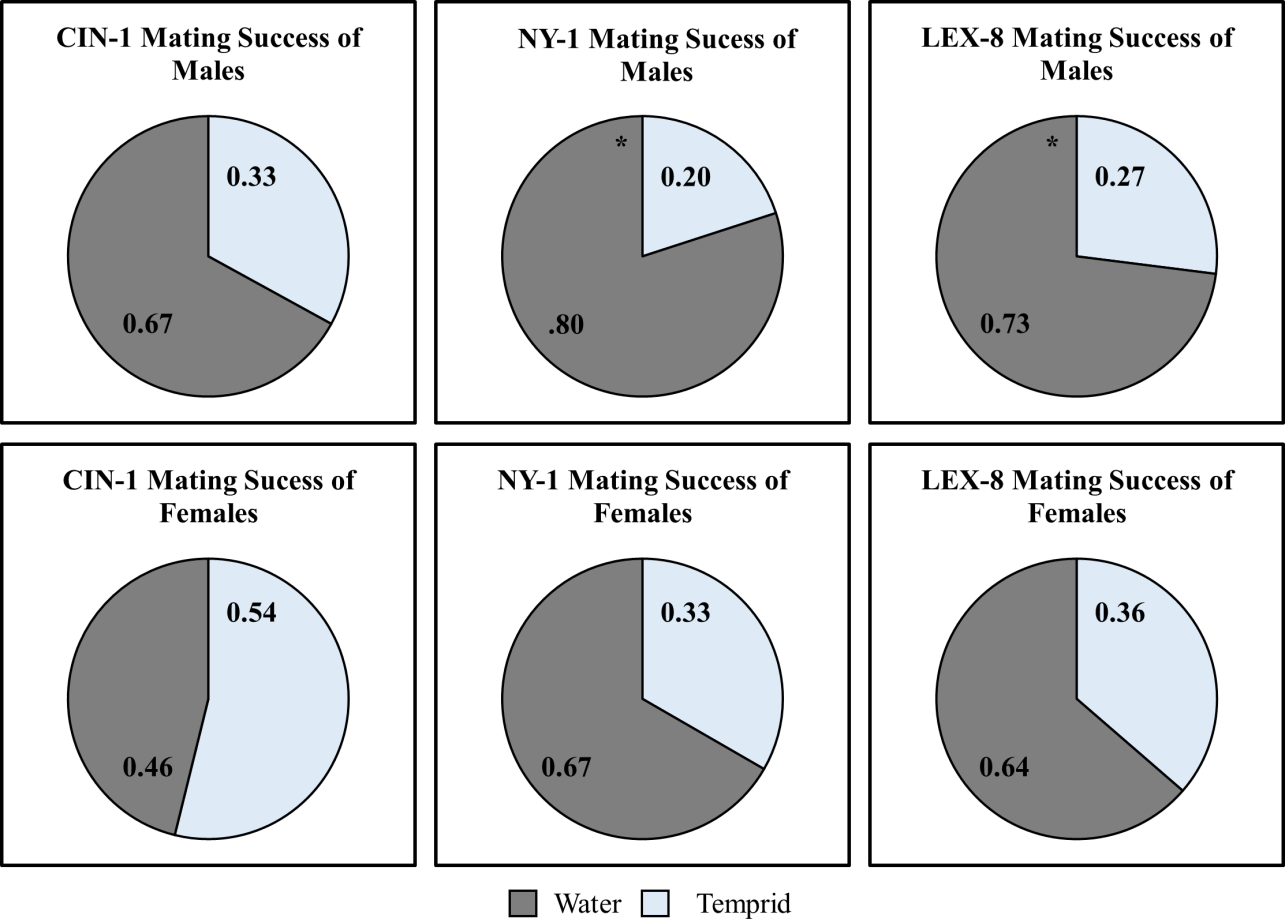
Mating success of treated and untreated males or females when an untreated member of the other sex is present. Males mated equally with treated and untreated females. However, when males had sublethal exposure (LT10)to the label rate of Temprid SC, there were significant reductions in successful mating events in the NY-1 and LEX-8 strains (Binomial Tests, n = 15, significant effects of treatment denoted with asterisks, *p<0.05). There were no non-responders.

For this experiment, we defined successful copulation (or a copulatory event) as prolonged mounting of a female by the male, with curving of the male’s abdomen. These behaviors are known to be associated with mating in this insect [18]. Using this definition, untreated males copulated with both treated and untreated females at equal frequencies in all three strains (Fig 1. Binomial Tests: CIN-1: p = 0.11, NY-1: p = 0.38, LEX-8: p = 0.70). However, when males from the moderately resistant and resistant strains were exposed to Temprid SC, they performed significantly fewer copulatory events on average (Binomial Tests: CIN-1: p = 0.15, NY-1: p = 0.05, LEX-8: p = 0.01). Sublethal exposure could impact the number of offspring a male produces if behavioral alterations lead to a reduction in mating attempts compared to his untreated counterparts. This is common; sublethal effects often reduce mating success in insect populations [33,34]. Insecticides can adversely affect male insects that rely on pheromones for mate-location, impacting their ability to detect sex pheromones and/or reducing behaviors crucial for mate finding—such as flight [33,34]. Unlike many other insects, the role of sex pheromones in bed bug reproduction is also ambiguous; no sex pheromone has been identified to date. It is possible, however, that a disruption in the ability of male bed bugs to detect alarm pheromone could lead to a reduction in fitness as superfluous homosexual mating attempts increase, since alarm pheromone has been shown to prevent these interactions [35]. This idea should be tested further by other researchers. However, because male bed bugs make multiple copulatory attempts over their lifetime [18], the impact of short-term reduction in these attempts may not translate into reductions in paternity or overall fitness.

### Fecundity with sublethal exposure before mating

Two different experimental designs were used to test whether sublethal exposure decreased the number of hatched offspring produced by male and female mating pairs after sublethal exposure. The first experiment relied on bed bugs that were selected shortly after adult emergence, while the second experiment used adults that emerged in isolation from other bed bugs. Therefore, in the second experiment, we could ensure that the insects were unmated before the experiment started. We did not expect results to change between the two designs, as other reports state that mating between teneral males and females is unlikely [30].

**Fig 2:**
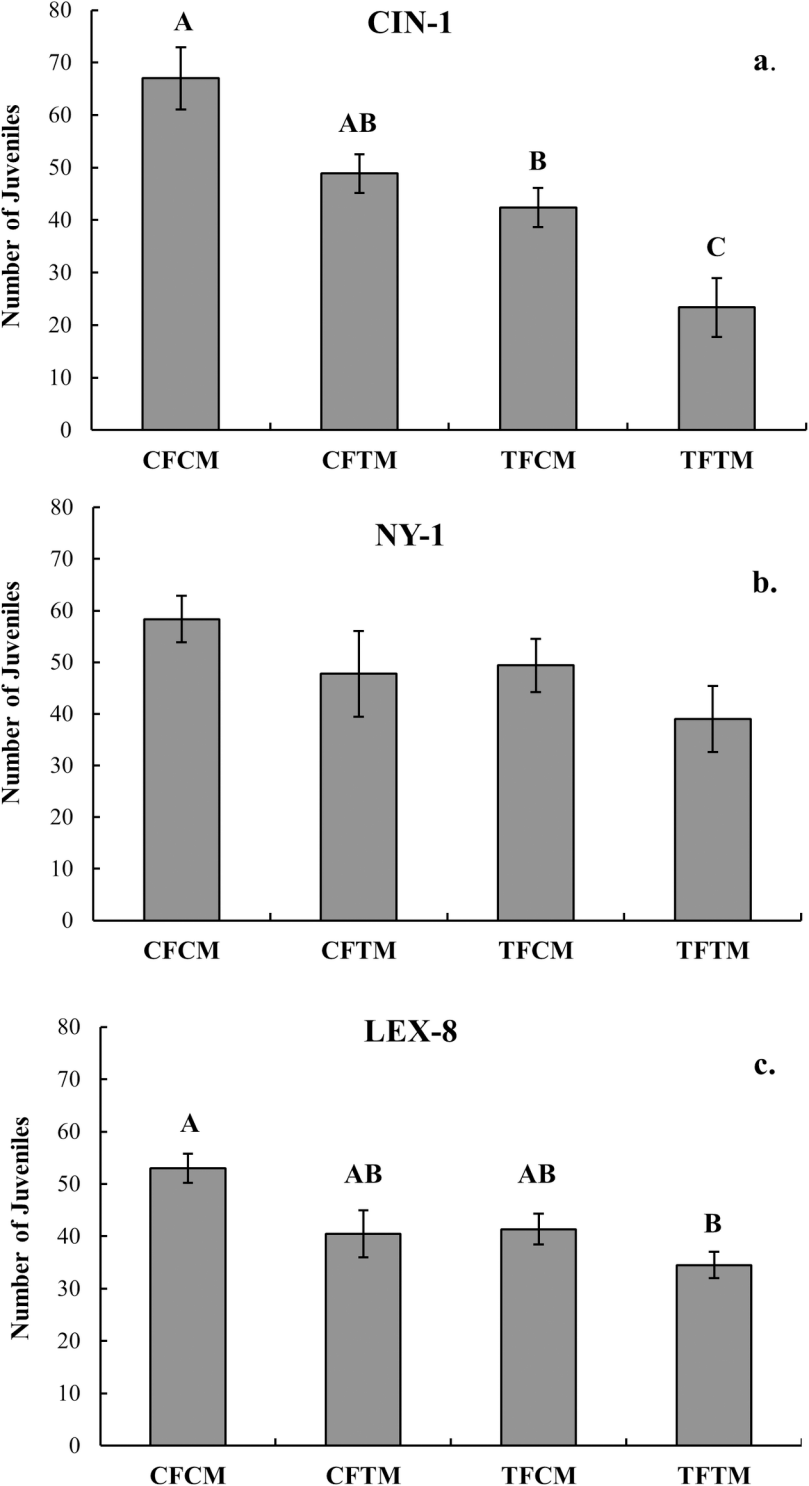
Characterization of successful egg hatch when sublethal exposure occurred before mate pairing. The number of hatched eggs (obtained by counting the number of juvenile bed bugs present) in all four combinations of treated and control males and females is presented (CF = Control Female, CM = Control Male, TF = Treated female, TM = Treated Male). Insecticide treatment via sublethal exposure (LT10) to the label rate of Temprid had a significant impact on the number of hatched eggs over the six week evaluation in two of three strains (ANOVA followed by Tukey HSD). When treatment groups share letters in common they are not significantly different (Tukey HSD test with α = 0.05). There was a steep decline in viable egg production when both male and female bed bugs in the mating pair had sublethal exposure (LT10) to insecticide, and an intermediate reduction in viable eggs when either the male or the female (but not both) experienced sublethal exposure. NY-1 (the moderately resistant strain) showed no significant reduction in the number of hatched eggs in this experiment.

In the first experiment, after six weeks, in two of the strains there was a significant reduction in the number of hatched eggs after insecticide exposure (Fig 2, CIN-1: F_3,28_ = 13.15, p<0.0001; NY-1: F_3,28_ = 1.66, p = 0.20; LEX-8: F_3,28_ = 4.73, p<0.01).In CIN-1, an independent impact of the insecticide on both males and females was apparent, because there was a significant drop in the number of eggs that hatched when both males and females were treated in comparison to when only one sex was treated (Fig 2A, 69% reduction in egg hatch). Similarly, in LEX-8, treatment of both males and females resulted in a decrease in the number of hatched eggs versus the control group (Fig 2C, 35% reduction in egg hatch). The overall trend was a sharp decline in the number of eggs that hatched when both male and female bed bugs were exposed to insecticide, and an intermediate reduction in hatched when either the male or the female (but not both) experienced sublethal exposure (Fig 2). Although not statistically significant, we observed the same trend for the moderately resistant strain (NY-1) as well.

**Fig 3:**
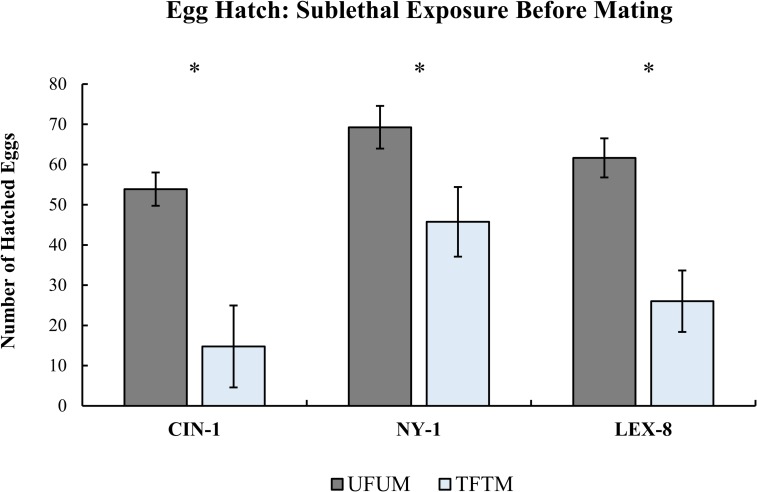
Characterization of the number of eggs hatched when sublethal exposure occurred before mate pairing. This experiment partially replicates the first fecundity test, but starts with males and females that had no previous contact with the other sex. There was significant reduction in the number of hatched eggs for every strain. Hatching decreased by 73%, 34%, and 58% for CIN-1, NY-1, and LEX-8, respectively, when both the male and female in the mating pair had sublethal exposure (LT10) to the label rate of Temprid SC prior to mating (Paired t-test, CIN-1: T = 3.59, df = 7, p<0.01; NY-1: T = 2.78, df = 7, p<0.05; LEX-8: T = 3.89, df = 7, p<0.01). Asterisks denote statistical significance (*p<0.05).

Due to our concern that rare mating events occurred between teneral males and females in the previous design, we repeated aspects of the experiment with bugs known to be virgin. Prior mating events before the experiment start could potentially confound results of the study. However, in this experiment there was also a significant reduction in the number of eggs hatched after six weeks between mating pairs in all three strains (Fig 3, Paired t-test, CIN-1: T = 3.59, df = 7, p<0.01; NY-1: T = 2.78, df = 7, p<0.05; LEX-8: T = 3.89, df = 7, p<0.01). The number of eggs that hatched decreased substantially, by 34%-73%, when both the male and female in the mating pair had sublethal (LT10) exposure to the label rate of Temprid SC prior to mating (i.e., the effect of sublethal exposure can be additive). Because the overall trend in both experiments was a sharp decline in the number of hatched eggs, we believe that sperm storage or previous mating events are not likely to change the results shown here. Mating pairs with sublethal exposure tend to consistently show stark drops in the number of viable eggs they produce, regardless of strain or mating status.

The mechanism for the reduction in the number of eggs that hatch after sublethal exposure is unknown, but could include physiological or behavioral factors [7]. One explanation is that females produce fewer viable eggs after treatment because they might imbibe less blood, which is used to produce eggs. Males may also reduce the amount of sperm they produce after sublethal exposure for the same reason. Alternatively (or in addition), because we did not observe the number of successful mating attempts in this assay, there is the possibility that male bed bugs were copulating less frequently after exposure. It should be noted, however, that in another study, researchers observed successful copulation, yet still recorded no egg production after sublethal exposure (1 or 10 minutes) to ActiveGuard™, a permethrin-impregnated fabric [27]. This result points toward the potential for sublethal effects on bed bug reproductive physiology [27]. More studies will be necessary to elucidate the mechanisms that are responsible for the decreased egg output in bed bugs with sublethal exposure to insecticides.

### Fecundity with sublethal exposure after mating

**Fig 4:**
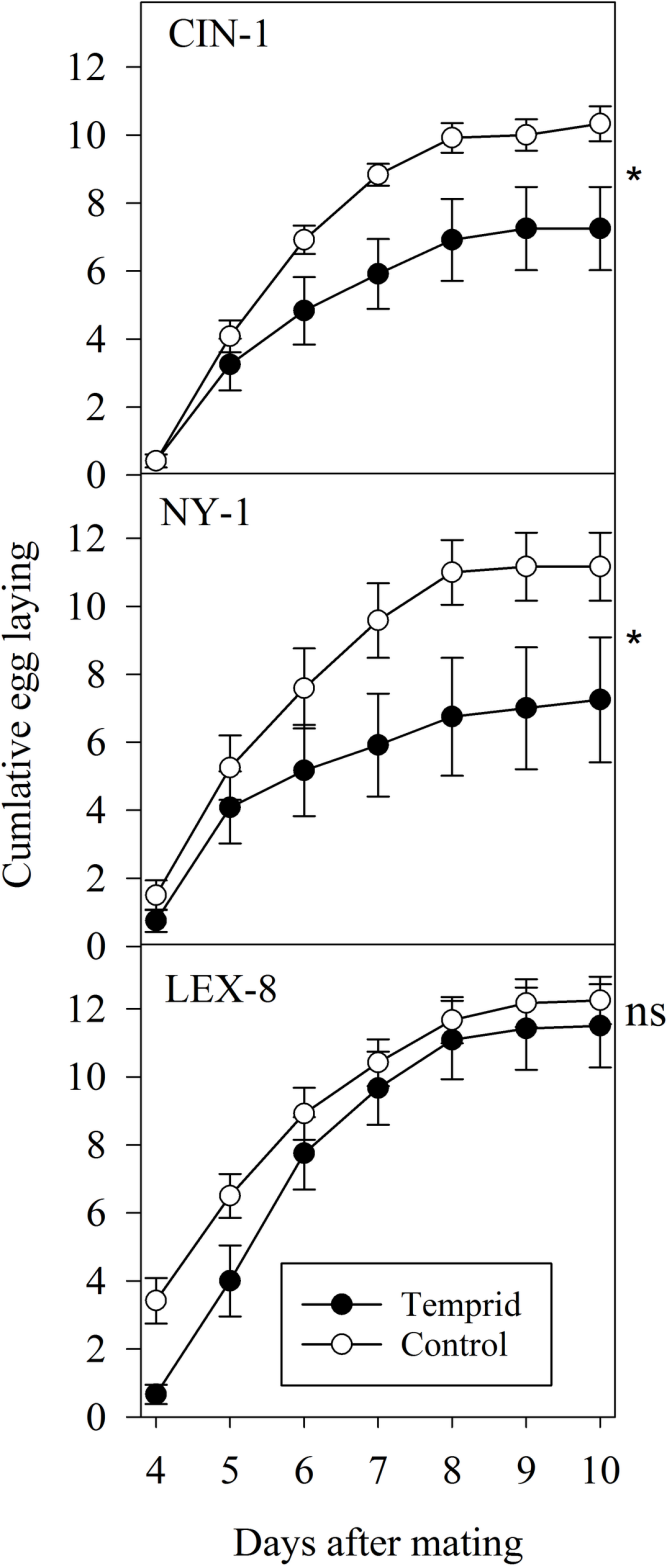
Cumulative egg laying for three strains when sublethal exposure occurred after mating had taken place. The total number of eggs laid significantly declined for the susceptible and moderately resistant strains (CIN-1 and NY-1), but not for the most resistant strain (LEX-8) (Paired t-tests, *p<0.05).

In this experiment, where we examined fecundity when the exposure to the insecticide occurred after mating, there were significant declines in the total number of eggs laid on average per female in all but one strain (Fig 4, Paired t-test, CIN-1: T = 2.01, df = 11, p<0.05; NY-1: T = 2.33, df = 11, p<0.05, LEX-8: T = 0.71, df = 11, p = 0.25). Females from the LEX-8 strain did not show a decline in egg production after treatment. The median day of egg laying for control strains was 5.67 ± 0.99, 5.75 ± 0.28, and 5.17 ± 0.24 days (mean ± s.e.m) for CIN-1, NY-1, and LEX-8, respectively. The median day of egg laying for treated bed bugs within the CIN-1 and NY-1 strains did not significantly differ from controls, with median days of egg laying at 5.67 ± 1.84 and 5.8 ± 1.73 days (mean ± s.e.m), respectively (Paired t-test, CIN-1: T = 0.00, df = 11, p = 1.0; NY-1: T = -0.23, df = 9, p = 0.82). However, the median day of egg laying for treated insects from the LEX-8 strain was significantly delayed compared to the control group at 5.92 ± 1.73 days (mean ± s.e.m) (Paired t-test, T = -2.35, df = 11, p<0.05) In addition, the first day of egg laying was significantly delayed for LEX-8, in opposition to the other two strains (Fig 4, Paired t-test, CIN-1: T = -1.41, df = 9, p = 0.19; NY-1: T = -1.62, df = 9, p = 0.14, LEX-8: T = -3.00, df = 11, p<0.05). The first day of egg laying for control LEX-8 females was 4.16 ± 0.11 days versus 4.92 ± 0.25 days for the treatment females.

The proportion of eggs that hatched significantly decreased in treatment groups for all three strains evaluated (Paired t-test, CIN-1: T = 2.62, df = 9, p<0.05, NY-1: T = 2.20, df = 8, p<0.05, LEX-8: T = 2.67, df = 11, p<0.05). The proportion of eggs hatched declined from 0.98 ± 0.01 to 0.76 ± 0.08 for CIN-1, 1.0 ± 0.00 to 0.86 ± 0.05 for NY-1, and 0.99 ± 0.01 to 0.91 ± 0.03 for LEX-8 (means ± s.e.m). This observation is consistent with the results obtained in the first two fecundity trials. It is apparent that females with sublethal exposure are capable of laying as many eggs on average, but those eggs may not be equally viable in comparison to control insects (as in the LEX-8 strain). Taken together, these data suggest that sublethal exposure will cause significant drops in egg hatchability whether insects are exposed before or after mating. Temporal patterns of egg laying may also shift, depending on the population of bed bugs.

The delay in oviposition demonstrated by the most resistant strain (LEX-8) should be investigated further for other strains of bed bugs. Oviposition delays could impact the timing of follow-up treatments in the field where a secondary treatment is sometimes applied to combat the hatching of newly laid eggs. Because LEX-8 was our most resistant strain, and thus received the longest duration of exposure to Temprid SC, it would also be worthwhile to explore whether other strains of bed bugs also delay oviposition when lengths of exposure to insecticide are extended. It is not uncommon for sublethal exposure to alter, and often delay, the pre and post-oviposition period in other insects [36].

### Development

**Fig 5:**
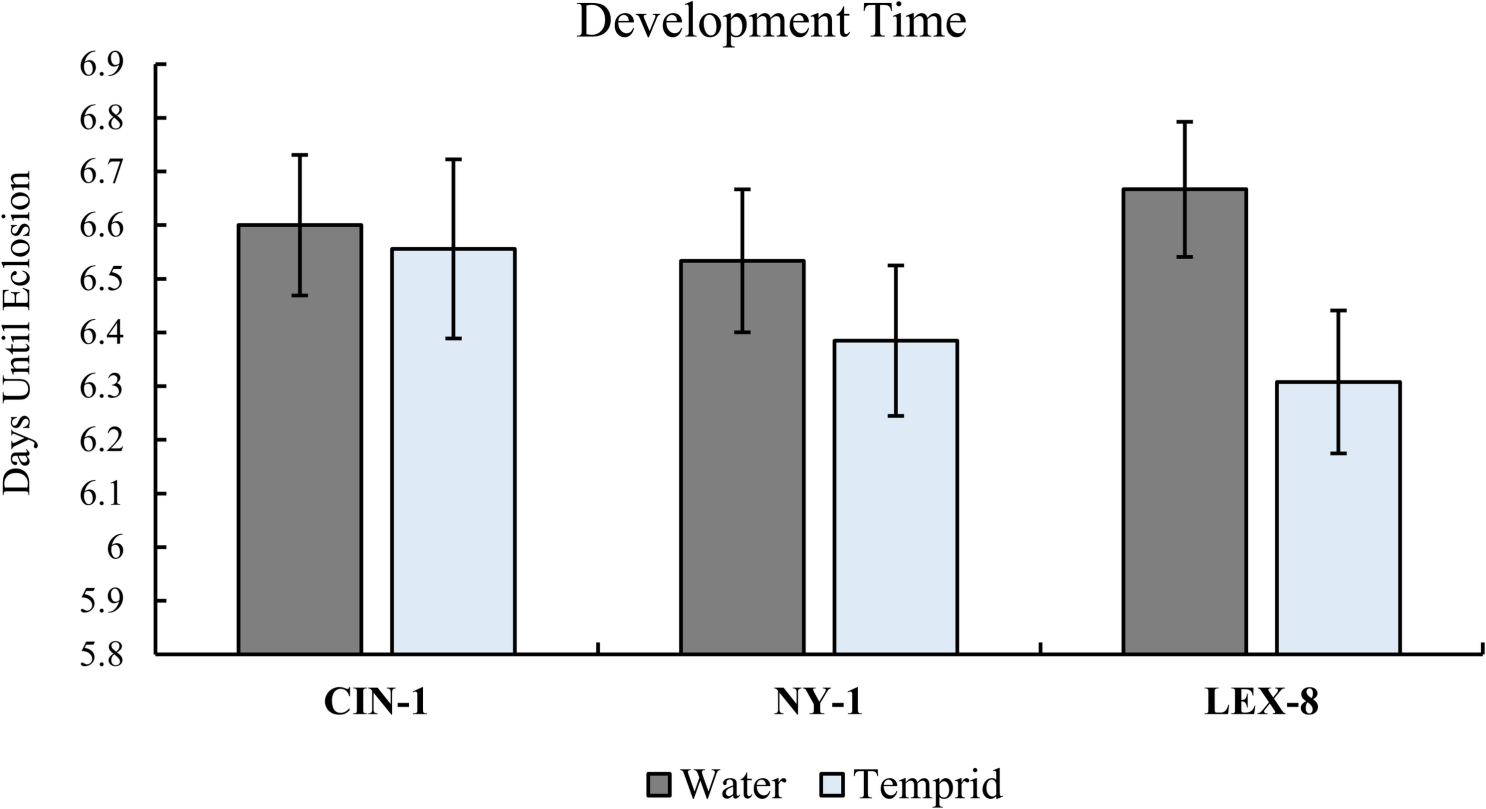
The time it took (in days) for fifth instars to molt to the adult life stage after sublethal exposure to Temprid SC. Fifth instars exposed to water served as a control. There were no significant differences within strain, and development time was not advanced or prolonged after sublethal exposure (Two sample t-test, CIN-1: T = 0.20, df = 22, p = 0.84; NY-1: T = 1.19, df = 26, p = 0.24; LEX-8: T = 1.96, df = 26, p = 0.06). There was mortality in the treatment groups (1 insect for CIN-1, and 2 insects for NY-1 and LEX-8), however the differences in mortality between the control and treatment groups were not significant (Fisher’s Exact Test, n = 15).

Contrary to our hypothesis, there was no delay in development time within the three strains we tested (Fig 5, Two sample t-test, CIN-1: T = 0.20, df = 22, p = 0.84; NY-1: T = 1.19, df = 26, p = 0.24; LEX-8: T = 1.96, df = 26, p = 0.06). In fact, on average, development time was slightly decreased for NY-1 and LEX-8 insects exposed to Temprid SC, however this effect was not statistically significant (Fig 5). There was some treatment mortality, but the difference was not significant when compared to controls (Fisher’s Exact Test, p>0.05). In some insects, sublethal exposure during a later instar can prolong development time until adulthood [37], however, development time was not significantly affected for bed bugs. It is possible that by selecting insects that successfully fed, we had already chosen a subset of insects that recovered more readily from exposure than other fifth instars. Additionally, observations more frequent than every 24 hours may have revealed more subtle differences in development time than we detected using this methodology. These differences would not likely be relevant for management purposes. However, future studies should address the recovery of juvenile bed bugs with sublethal exposure to insecticides, and whether they are as likely to take blood meals as control insects. This would impact the number of emerging adults in an infestation.

## Conclusions

We designed this study to more thoroughly evaluate one popular combination product used against bed bugs, Temprid SC, to determine whether sublethal effects had the potential to influence bed bug populations or their management. We hypothesized that sublethal exposure would have detrimental effects on individual bed bugs. These effects could have population-level consequences if multiple individuals display decreased mating success, decreased egg output/fecundity, and longer maturation time after sublethal exposure. In this study, we saw changes in mating success and fecundity of bed bugs after sublethal exposure to Temprid. We saw no changes in development time or success for fifth instars, but effects on development of juveniles should be characterized further due to the slight decrease in development time observed in two populations. Nonetheless, the overall detrimental effect of sublethal exposure (LT10) to the label rate of Temprid SC on bed bug fecundity may contribute positively to bed bug management if many individuals are affected. However, the potential for recovery from sublethal effects needs to be explored in depth before decisions are made based on our results (such tests are currently in progress). Laboratory studies of development and behavior such as those reported here should be interpreted with caution because of potential differences between laboratory (e.g., rabbit blood presented with an artificial membrane) and real world conditions (e.g., a human host and more complex habitat). Nonetheless, insecticide efficacy testing that includes the evaluation of sublethal effects on behavior, development and fecundity, when carefully controlled, can complement traditional mortality based assays. Incorporation of these assays during efficacy trials may help to explain the occasional incongruous results between acute toxicity and field efficacy.
